# Resurrection of the genus *Staphisagria* J. Hill, sister to all the other Delphinieae (Ranunculaceae)

**DOI:** 10.3897/phytokeys.7.2010

**Published:** 2011-11-29

**Authors:** Florian Jabbour, Susanne S. Renner

**Affiliations:** 1 Systematic Botany and Mycology, University of Munich (LMU), Menzinger-Str. 67, 80638 Munich, Germany

**Keywords:** *Aconitum*, *Delphinium*, Mediterranean region, molecular phylogeny, nomenclature, *Staphisagria*

## Abstract

Molecular sequence data show that the three species o*Delphinium* subg. *Staphisagria* (J. Hill) Peterm. form the sister clade to *Aconitum* L., *Aconitella* Spach*Consolida* (DC.) S.F. Gray, and all remaining species of *Delphinium* L. To account for this finding we resurrect *Staphisagria* J. Hill (1756). Names in *Staphisagria* are available for two of the species. We here make the required new combination for the third species, *Staphisagria picta* (Willd.) F. Jabbour, provide a key to the species, and illustrate one of them.

## Introduction

*Delphinium staphisagria* L., *Delphinium requienii* DC., and *Delphinium pictum* Willd. are annual or biennial species of the tribe Delphinieae (Ranunculaceae) that occur in the Mediterranean basin (see ‘Nomenclature and key to the species’ section for a more detailed description of their distribution areas). They are considered endangered (Olivier et al. 1995, Fraga et al. 2004) because of changing land use patterns and bottlenecks caused by irregular demography (Orellana et al. 2009a). All three species are protected in France (Olivier et al. 1995).

Linnaeus knew only *Delphinium staphisagria*, which he described as *Delphinium nectariis diphyllis*, foliis palmatis lobis integris. With the recognition in the early 19^th^ century that there were two additional species resembling *Delphinium staphisagria*, Spach (1839) grouped all three in the genus *Staphisagria* established by John Hill in 1756 for Linnaeus’s *Delphinium staphisagria*. Spach’s ranking of the three species as a separate genus, distinct from *Delphinium*, however, gained few followers and no modern treatment appears to have accepted *Staphisagria*.

**Figure 1.**
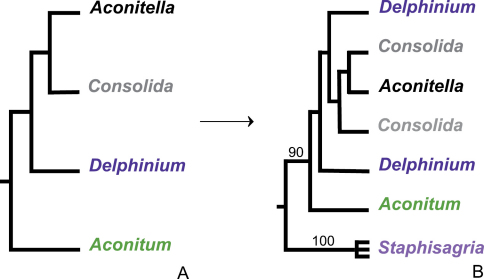
Schematic representation of the phylogenetic relationships in the Delphinieae **A** as suggested by studies anterior to the findings of Jabbour and Renner (unpublished data) and **B** as found with combined nuclear (ITS) and plastid (*trn*L intron and adjacen*trn*L-*trn*F intergenic spacer) DNA sequences (2088 aligned nucleotides) from 191 accessions representing 185 species of Delphinieae (Jabbour and Renner, unpublished data). In this study, taxon sampling covered all genera and subgenera of the tribe. Numbers above branches indicate Maximum Likelihood bootstrap supports.

Molecular phylogenetic studies of the Delphinieae recently revealed that the three species of *Staphisagria sensu* Spach are the sister clade to all other Delphinieae (Maximum Likelihood bootstrap support: 90%), a group of 650-700 species ranging from Eurasia into North America and with a few isolated species on West and East African mountains (Jabbour and Renner, unpublished data; Fig. 1). This discovery fits with several characters of the three species that are unusual in *Delphinium*. For example, *Staphisagria* species have eight chromosome pairs of staggered size (see Fig. 3 in Verlaque and Aboucaya 2001), while most *Delphinium* have a bimodal karyotype of two long and six short chromosome pairs (Gregory 1941; Kurita 1955; Blanché and Simon 1987; Yang 1996, 2001). Species of *Aconitum* subg. *Lycoctonum* (c. 50 species) and the three species of *Staphisagria* (Verlaque and Aboucaya 2001) have a similar karyotype, suggesting parallel chromosomal reconfigurations. The *Staphisagria* species also have C19 aconitine-type alkaloids (De La Fuente and Reina 1990) and *Aconitum*-like stomata and pollen (Blanché 1991). Flowers of *Staphisagria* are less zygomorphic than those of the remaining Delphinieae and their nectar spurs are only 2-7 mm long (Bosch 1997; Verlaque and Aboucaya 2001). This last trait probably reflects predominant self-fertilization, with reduced reliance on nectar-foraging bees for cross-pollination (Bosch 1997; Bosch et al. 2001).

To account for the phylogenetic relationships in the Delphinieae (Fig. 1), we here resurrect the genus *Staphisagria* and make the required new combination for a species for which Spach (1839) did not provide a legitimate name.

## Nomenclature and key to the species

### Staphisagria

J. Hill, Brit. Herbal: 44. 1756.

#### Type.

*Delphinium staphisagria* L., Sp. Pl.: 531. 1753 [original type, cited by its nomen specificum legitimum].

### Key to the species

**Table:** 

1	Spur of the upper tepal 1/5-1/3 as long as perianth segments (Fig. 2C); seeds 5.5-7.5 mm (Fig. 2F)	*Staphisagria macrosperm*a
–	Spur of the upper tepal at least 2/5 as long as perianth segments; seeds 3–4.5 mm	2
2	Inflorescence axis, pedicels, and outside of perianth segments shortly pubescent; bracteoles inserted at the base of the pedicels	*Staphisagria picta*
2	Inflorescence axis, pedicels, and outside of perianth segments villose-hirsute; bracteoles inserted at some distance above the base of the pedicels	*Staphisagria requienii*

### Staphisagria [“ Staphysagria ”] macrosperma

Spach, Hist. Nat. Vég. 7: 348. 1839.

#### Replaced name.

*Delphinium staphisagria* L., Sp. Pl.: 531. 1753. TYPE: *LINN 694/12*, Habitat in Istria, Dalmatia, Calabria, Apulia, Creta, Galloprovincia [South France]. The geographic origin of Linneaus’s type cannot be narrowed down (Munz 1967, Ilarslan 1996, Jarvis 2007).

#### Herbarium specimen studied:

Greece: Crete, Nomos Lassithiou, ravine between Zákros and Kato Zákros, 70 m, 15 May 2002, E. Vitek 02-205 (W, GZU, M, MA).

#### Comments

**Figure 2.**
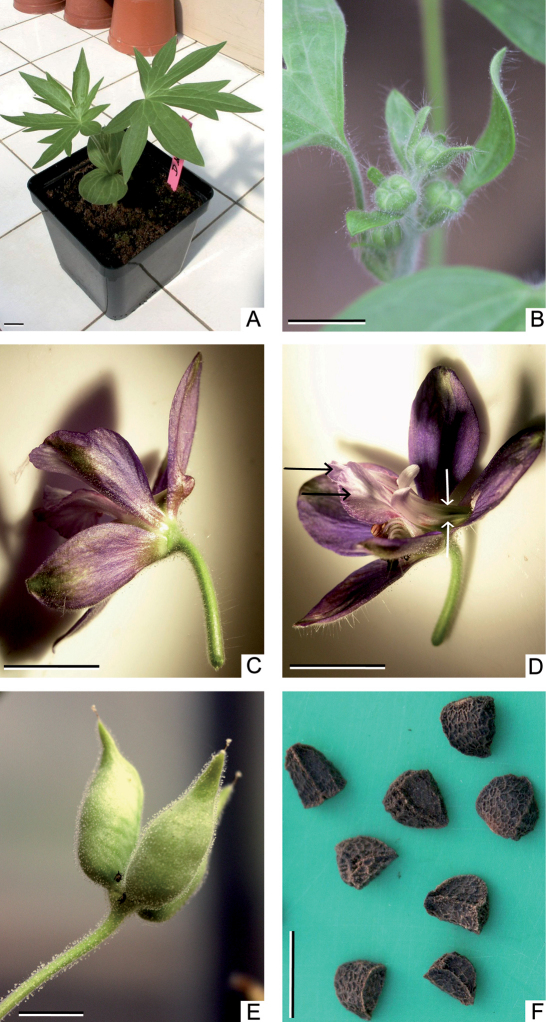
*Staphisagria macrosperma* **A** young plant with the cotyledons and two palmate leaves **B** young inflorescence with floral buds subtended by a bract and bracteoles **C** side view of a flower showing the very short spur (or bulge) on the dorsal petaloid tepal **D** three-quarter view of a flower showing four developed staminodes: two lateral (black arrows) and two spurred (white arrows). The tips of the spurs are nested within the bulge of the dorsal tepal **E** three follicles **F** Gravity-dispersed poisonous seeds (c. 6 mm in length). Scale bars: 1 cm.

Spach had to chose a new name for this Linnaean species because *Staphisagria staphisagria* would be an exact tautonym (not permitted in botanical nomenclature), and since his misspelling of Hill’s genus (as *Staphysagria*) is a correctable error (variant spelling), Spach’s name *Staphisagria macrosperma* is legitimate. Among the three species of the genus *Staphisagria*, *Staphisagria macrospermahas* the largest distribution. Because of its ancient use in medicine (Cristofolini and Mossetti 1998), it is found all around the Mediterranean basin (Greuter et al. 1989; Orellana et al. 2009a). It grows in rocky areas, and is adapted to nitrophilous and disturbed habitats (Orellana et al. 2009a). Figure 2 shows key morphological characteristics of *Staphisagria macrosperma*.

### Staphisagria requienii

(DC.) Spach, Hist. Nat. Vég. 7: 350. 1839.

#### Basionym.

 *Delphinium requienii* DC., Fl. Franç. (DC. & Lamarck), ed. 3. 5: 642. 1805.

#### Herbarium specimen studied:

France: Var, Hyères, Porquerolles island, 12 Jun 1961, Gavelle s.n. (M).

#### Comments

*Staphisagria requienii* is a narrow endemic of the Mediterranean Archipelago of Hyères, Var, South of France (Verlaque et al. 1991). It grows in a variety of habitats, like crops, calcareous rocks, and degraded areas along roads (Orellana et al. 2009b).

### Staphisagria picta

(Willd.) F. Jabbour, comb. nov.

#### Basionym.

*Delphinium pictum* Willd., Enum. Pl. [Willdenow] 1: 574. 1809. SYNTYPES: Röpert, D. (Ed.) 2000- (continuously updated): Digital specimen images at the Herbarium Berolinense. Published on the Internet http://ww2.bgbm.org/herbarium/ Barcode: B -W 10324 -01 0 / ImageId: 164585) and Barcode: B -W 10324 -02 0 / ImageId: 164601) [accessed 02-Sept-11].

#### Herbarium specimen studied:

Balearic Islands: Majorca, Puntas de Covas, top of sea cliffs, amongst limestone boulders, 100 m, April 1988, F.J. Rumsey s.n. (M).

#### Comments

The new combination is necessary because *Staphisagria brevipes* Spach (1839) is illegitimate since it included the older name *Delphinium pictum*. *Staphisagria picta* is endemic to Corsica, Majorca, and Sardinia. Its main habitats are open grasslands covering rocky places from 150 up to 600 m above sea level (Orellana et al. 2009b)

## Supplementary Material

XML Treatment for Staphisagria

XML Treatment for Staphisagria [“ Staphysagria ”] macrosperma

XML Treatment for Staphisagria requienii

XML Treatment for Staphisagria picta
